# New York Odontological Society

**Published:** 1890-12

**Authors:** 

**Affiliations:** New York Odontological Society.


### NEW YORK ODONTOLOGICAL SOCIETY.

THE New York Odontological Society held its regular monthly meeting Tuesday evening, October 21, 1890, in the New York Academy of Medicine, No. 17 West Forty-third Street. The President, Dr. J. Morgan Howe, in the chair.

*The President.*—Since the last meeting of this Society, one of our oldest and most highly esteemed members has passed away. Dr. William A. Bronson, who died on the 20th of last August, had for many years been an honor to dentistry. He stood in the front rank by reason of his intellectual and manipulative ability, and his genial, kindly nature endeared him to all who knew him. It seems appropriate, and undoubtedly is the wish of the Society, to take some action in regard to our loss.

*Dr. Lord.*—I move you, Mr. President, that the Chair appoint a committee of three to draw suitable resolutions in regard to the death of Dr. Bronson.

The motion prevailed, and the president appointed as a special committee Drs. C. E. Francis, Charles Miller, and S. G. Perry.

*The President.*—I have to announce to the Society that, in accordance with the recommendation of the Executive Committee, whose report was adopted at the last regular meeting, the Chair has appointed a committee of this Society to confer with committees of other societies in relation to the proposition for the establishment of a Dental Club, such committee being composed of Drs. Bödecker, Carr, and Jarvie. The gentlemen named have been notified by the secretary of this Society of their appointment. It is hoped that the committee will meet with other committees that may be appointed by the other societies interested, and that we may hear from them later, not in the Society but through other channels.

*Dr. Jarvie.*—Who is to take the initiatory steps of such a meeting?

*The President.*—It would be very proper, I think, for the committee appointed from this Society to communicate the fact of that appointment to the other societies and request the appointment of committees to meet with them and confer together for the elaboration of a scheme of organization. The recommendation of our Executive Committee did not make any special provision for that, but I think it would be appropriate for our committee, as the first appointed, to make such a communication to the other societies.

I have received a letter from Dr. J. D. Miles, of Vicksburg, Miss., the purport of which is, that he wishes to present a gavel to the New York Odontological Society, as a token of the high esteem he has for the Society. This gavel is made of wood that has historic interest. It is made of a portion of the steamer "Star of the West," which carried Walker and his filibusters to Nicaraugua. It was the first vessel chartered by the United States government in our recent civil war; it was the first vessel fired upon by the Confederates; and it was the first vessel in the service of the United States government captured by the Confederates. It was afterwards sunk, and has remained under water all these years; recently a part of it has been raised, and Dr. Miles has had this gavel made of a part of that wood.

On motion of Dr. Francis, the gavel was accepted with the thanks of the Society.

Dr. Kingsley offered the following preamble and resolution:

WHEREAS, There are a number of gentlemen in the profession who, beginning practice about the year 1840, were acquainted with the early pioneers of dentistry. As these gentlemen are approaching or have reached the fiftieth anniversary of their dental career, it would seem that it would be a courteous and proper thing to tender them altogether a banquet. Therefore, be it

*Resolved,* That this Society appoint a committee of three to co-operate with similar committees appointed by the local societies hereabout in undertaking and providing such a banquet as above proposed.

The resolution was passed, and the President appointed as such committee Drs. Kingsley, Perry, and Mirick.

*Dr. S. C. G. Watkins.*—Mr. President, I have a communication from the Central Dental Association of Northern New Jersey, which I would like to present to this meeting. I was appointed a committee to come here this evening and present to your Society a matter of great interest to all American dentists. On the 15th of September, before the Central Dental Association of Northern New Jersey, a paper was read by Dr. Rehfuss, of Philadelphia, the purport of which was a description of the nature of patents. In that paper he drew a distinction between patents upon operations in the mouth and patents upon instruments, elaborating the subject quite extensively. The result of the paper was that our society appointed a committee to bring this matter before the dental profession of America, and urge its societies to co-operate with them for the purpose of getting a bill passed by Congress to prevent the issuing of patents upon operations in the mouth. Of course you all know the idea of that is to prevent further monopolies of patents on operations in the mouth, which will retard our work in our profession. I will read you the papers, which have been prepared by a lawyer of considerable ability in New Jersey. I will say that I presented this matter to the First District Society last week, and a committee of three was appointed by that Society to co-operate with other committees. It is to be hoped that every dental society in America will take this matter up immediately and appoint committees to co-operate with us in this good work so that we may be able to get it through Congress this winter, if possible. At present all communications should be sent to Dr. William L. Fish, Newark, New Jersey.

[Dr. Watkins read the petition and the draft of the proposed act.]

*Dr. Allan.*—Is it possible for Congress to pass an *ex post facto* law annulling patents that have been granted?

*Dr. Watkins.*—This bill was drawn by an able lawyer of Newark.

*Dr. Littig.*—I move you that a committee be appointed to confer with the committees already appointed.

*Dr Jarvie.*—Mr. President, that paper speaks of an expense of about one thousand dollars. I would be opposed to the appointment of a committee if there is any expense attached to it, for this reason, that I do not think any such bill would be likely to be passed by the House of Representatives.

*The President.*—I do not suppose that the appointment of a committee to confer would involve any expense.

Dr. Littig's motion prevailed, and the President appointed under it, Drs. J. B. Littig, George S. Allan, and S. E. Davenport.

*Dr. George S. Allan.*—Mr. President, if it is proper to do it now, I would like to make a request of our members and others. I have been engaged for some time in some studies of pyorrhœa alveolaris, and I very much want specimens of teeth that have been extracted because of this disease, with any brief statement of the conditions and the treatment that may have been adopted. There are two widely divergent theories respecting the trouble, and there is no way of getting at the bottom facts in relation to them except by careful examinations of many specimens, and those specimens must be accompanied with some little history as to the duration of the disease, the condition of the mouth, and the treatment that may have been adopted. I will take it as a great favor if any gentleman who may be called upon to extract teeth for pyorrhœa alveolaris will let me have such teeth for microscopic and other examination.

*Dr. Jarvie.*—What does the gentleman mean by two divergent theories?

*Dr. Allan.*—One theory is that pyorrhœa alveolaris is of systemic origin entirely. The other is that a local fault, that may or may not have a constitutional origin, is the principal factor in producing the disease. One class of thinkers say that the disease can have its inception and its progress without any mechanical irritation whatsoever in the mouth, and others say that mechanical irritation must precede the disease itself. I read a paper on the subject before the New Jersey State Dental Society a year ago last July, I think it was, and since then I have been carrying on my studies and investigations. Of course every dentist is limited in his practice as to the number of teeth he can obtain, and I want to enlarge my material and obtain more specimens for this express purpose.

*The President.*—Will it not be well for Dr. Allan to allow his address to go into our published report, so that dentists reading his request may know where to send specimens?

*Dr. Allan.*—My address is 51 West Thirty-seventh Street, Dr. George S. Allan.

*Dr. Littig.*—Mr. President, I had an interesting case just before I left town this summer, that presented some peculiar conditions. I have always believed that the abrasion which we find upon the teeth was more or less mechanical, where it was not associated with decay, but a case came into my hands wherein the abrasion or wasting away of the enamel was equal both on the labial and the palatal surfaces, and the teeth had become so thin that nothing could be done with them in the way of filling. The labial surfaces of the front teeth were discolored, but the palatal surfaces were perfectly clean, white, and smooth. I relate this merely to describe the treatment or mechanical appliances I adopt in the restoration of this class of teeth. In this case I covered the teeth with thin platinum and gold caps, making the caps to fit the teeth as closely as I could; first making a band to fit around the cervical margin of the tooth, then slitting it down while in the mouth, and bending it to fit the tooth perfectly. On the ends of these bands I soldered a little strip across, making a box; then took pieces of porcelain, and ground them thin, and put faces over these gold caps, soldering the face to the cap, and set them with zinc-phosphate. On the centrals and laterals I worked in that way, and on one of the bicuspids and the cuspid I made the boxes slightly different. It is a method suggested by Dr. Schofield. I made the caps similar to the ones I just spoke of, but instead of soldering the porcelain directly upon those caps I made a horseshoe band down towards the necks of the teeth, slipped it up towards the proximal edges, then put a piece across the end of it, and cemented a piece of porcelain on to this little box, —for the purpose of giving strength for masticating. I accomplished the setting of these caps just before I left town, and I had the pleasure of seeing them recently, and finding them all intact, and doing good service, with one exception, and that was one of the cemented pieces of porcelain; the cement had not held it as firmly, or I had not lapped the edges quite as well as I ought, and it became loosened. I would like to know if there is any other way of treating such cases satisfactorily. The pulps in these teeth are all alive; and to cut the teeth off, destroy the pulps, and put on crowns seemed to me to be a sacrifice that I did not want to have the patient make.

*Dr. Watkins.*—Mr. President, Dr. Littig, speaking of abrasion, reminded me of a case I had lately, a young lady, nineteen years of age, with quite an extensive abrasion on the superior central, lateral, and bicuspid of the right side, and the cuspid and first bicuspid of the lower jaw. I never saw a case like it before in a person of that age, and it strikes me that such a case must certainly be chemical rather than mechanical. I want to relate another case, and ask for information. A lady came to my office a few weeks ago, saying she had been to different dentists throughout the country, and almost every time she had consulted a dentist he had found a tooth with a dead pulp; in almost every case where a filling had been taken out for the purpose of refilling, the tooth was found to be dead. The pulps had died without giving any pain, or any outward appearance of trouble. I found four such teeth in her mouth, and I think there were five or six before. There was no pain from them. The fillings that were put in previously were not very large fillings, and did not approach closely to the pulps, and yet the pulps died.

*Dr. Littig.*—Do you know the history of the family?

*Dr. Watkins.*—I do not. The lady is enjoying perfect health, apparently.

*Dr. Winkler.*—I saw two or three cases, some time ago, in which pulps died from very slight irritation, and I thought they were due to some hereditary trouble.

*Dr. Littig.*—I saw a case similar to the one Dr. Watkins has spoken of, but I learned, afterwards, that the patient had been in the hands of a man who was using a kind of paste, containing arsenic, for obtunding sensitive dentine. The teeth were filled, and did not give much trouble until after the fillings were removed. I wonder if the case Dr. Watkins has related had not something of that kind in its nature,—whether arsenic had not been put in the lady's teeth for a short time for the purpose of obtunding sensitive dentine.

*Dr. Watkins.*—I think that would be hard to find out without getting at the dentist; and probably he would not tell.

*The President.* If there is nothing further under the head of Incidents of Office Practice I will call upon the first essayist of the evening to present his paper. Dr. George A. Wilson, whom I need not introduce to you, will read a paper on "The Treatment of Proximate Surfaces." (For Dr. Wilson's paper, see page 732.)

*The President.*—Dr. George H. Winkler has a paper on the same subject, and I think we will hear it before we enter upon the discussion. (For Dr. Winkler's paper, see page 729.)

*The President.*—Gentlemen, the subject treated by the papers presented to you is now before you for discussion.

*Dr. George S. Allan.*—Mr. President, I am astonished that a dozen gentlemen do not jump up at once to discuss this interesting subject, a subject that we have all thought of a good deal, and that is the A, B, C, almost, of our profession. I came here to-night hoping to hear something upon the principles governing the business of putting in contour fillings. The whole line of thought that is mapped out in Dr. Wilson's most admirably-written paper, is old, old as the hills. We all know it; we have it by heart; and axioms and truisms we do not want. I have listened to them until I am tired of them. It is almost an insult to tell a dentist of ability and character, who wants to do the best work, that contour fillings are the best. He knows it. What is the use of going back and rehashing and giving us chestnuts *ad nauseam.* I would give more to have some one offer me a new instrument, or tell me a new fact as to the structure of a tooth, or the physical qualities of gold and how to adapt gold to a tooth, or how to overcome the difficulties that I am constantly meeting, than to hear these old things talked over from time to eternity.

Dr. Perry, in an admirable paper, has told us why we should put in contour fillings, and has done it so thoroughly that to-day it stands probably as the best monograph on the subject extant. But Dr. Perry for me has done more: he has put into my hands, lately, his modification of the Bonwill mallet, a beautiful instrument, and with it I can overcome difficulties that I could not overcome before. It is worth more to me as an argument in favor of contour fillings than a dozen of his essays would be.

In that celebrated work that you have all read, the "Pickwick Papers," I think that it is Winkler who asks Pickwick at an election, where he is a good deal confused as to what to do, "Which crowd shall I shout with?" and Pickwick, with that wonderful wisdom for which he was celebrated, said, "Shout with the largest." Now, gentlemen, it may be prudent to shout with the largest crowd, but the largest crowd is not always the wisest. The truth is, in this case, the largest crowd keeps silent. They do not covet abuse and criticism. They are eclectics in practice, and mostly elect to put in face fillings. Dr. Wilson alluded in his paper to a simple statement of fact which I made. It contained no principle to endorse, no practice to advocate. I simply stated a fact which stands undisputed: that was that the bulk of the fillings of the day were face fillings, and the theory of the day was contour fillings. Then I ask the question, Why is this so? Can we solve the riddle? Why is it that dentists are incessantly talking contour fillings, and practising face fillings? I gave some reasons in the paper I read why contour filling was practised with difficulty, more difficulty than face filling, and I tried to show why we should act with the wisdom of a general before his enemy, and acknowledge these difficulties. The general who in war belittles his opponent, and pays no attention to what his numbers or armaments may be, is a foolish man, and is very apt to be defeated. So Dr. Wilson's conclusions in regard to my statement have no force or point, and require no answer. The whole purport and aim of my paper was to ask you to look this thing in the face. We all want to put in contour fillings; we know they are the best; we are constantly aiming to put them in well and thoroughly; but how shall we do it? What new knowledge do we want, what new instruments do we need, to help us realize more fully our ideal? I was criticised most violently for writing that paper. One man said I was forty years behind the times; another that I was making a bid for face-filling work; but not one single argument in the paper was answered, not one statement of fact controverted. Therefore I do not feel that any of the criticism passed upon me was just or fair. What I want is to have this subject approached from an entirely different stand-point from what it has ever been approached before. I want to know what is the philosophy and the science of it; I want to know what the best instruments are; I want some downright instruction; and I think that is what we are all aiming at. But I reiterate what I know to be a fact, and what every one present knows to be a fact, that while contour fillings are most desirable, yet face fillings are in the majority.

*Dr. Jarvie.*—Mr. President, Dr. Allan says he is seeking some new philosophy which will help to solve the riddle of why we all advocate contour fillings, yet in practice so many face fillings are put in. I think it is a matter that is very easily solved. Every gentleman in this room will agree with me that the most perfect dentures are those in which the teeth impinge near the grinding surfaces, or cutting edge, leaving a space at the margin of the gum, a V-shaped space, so that any small particles of food that may be forced between the teeth may pass out freely and readily where the space is largest. That, I think, is our ideal of the most perfectly-formed set of teeth. We do not find them in every mouth; but it follows, naturally enough, that the nearer we can restore broken or decayed teeth to their perfect type the better it is for the teeth. Now, if that is the case, why don't we always do it? The reason is, that while contour fillings are by far the most desirable, it would often be most unwise to make them; the operator finds the conditions such that it is impossible to make a perfect contour filling, while, under these same conditions, he can make a perfect face filling; and a perfect face filling is better than an imperfect contour filling.

*Dr. Allan.*—You are forty years behind the times!

*Dr. Jarvie.*—Nevertheless, a perfect face filling is better than an imperfect contour filling. Sometimes the condition of the patient is such that we are not warranted in making contour fillings. The class of people who have the frailest teeth are those that are the least able to stand the prolonged strain necessary to the making of contour fillings, and in such cases it would be unwise, even if it were possible, to make a weak and delicate patient submit to any tedious and trying operation. It is far better for them to have fillings renewed from time to time, than to subject their nervous system to any such barbarity as a gentleman told us about some time ago, of keeping a patient in the chair six hours at one sitting while making a filling. So I think it is very easy to account for the fact that while we hold to the theory of contour fillings, our practice at times is quite different.

This, Mr. President, is a most interesting subject, the subject of the proper treatment of approximal surfaces; in fact, it involves almost an entire treatise on operative dentistry. Almost every other surface of the teeth, with the exception perhaps of the buccal surface, where cavities run far under the gum, can be easily cared for, and requires comparatively little skill; but there is such a variety and multiplicity of conditions that have to be taken into consideration in treating these approximal surfaces, that we may make mental reservations in discussing the subject that our listeners do not appreciate or understand, and therefore our statements are misunderstood. It is a subject that covers a great deal of ground and that cannot be treated properly in a paper requiring ten or fifteen minutes to read.

*Dr. Allan.*—Mr. President, I am exceedingly glad to listen to Dr. Jarvie. He has taken precisely the position that I take, and advocates precisely that which I advocate. Now, I think that we can only approach this subject from the stand-point of physics and of law. If we can get instruction how to do our work and better our operations, we will all be very much benefited. I confess that I felt sore over the reception of the paper which I read.

The idea that I did not advocate contour fillings, and that the paper was written in advocacy of face fillings, was surprising to me, and how any one who listened to the reading of it could gather any such misinterpretation I cannot conceive. I do not believe there is any one in the Society who has advocated or does advocate contour fillings more emphatically than I do, or who more constantly practises the putting in of that class of work. I do hope somebody will take up this subject and discuss it from the stand-point I have indicated.

Adjourned.

S. E. Davenport, D.D.S., M.D.S.,
*Editor New York Odontological Society.*

